# Thermogenic tissues in lotus: Insights from multiscale imaging and calcium dynamics

**DOI:** 10.1093/plphys/kiaf173

**Published:** 2025-05-02

**Authors:** Erin Cullen, Chong Teng

**Affiliations:** Assistant Features Editor, Plant Physiology, American Society of Plant Biologists; Assistant Features Editor, Plant Physiology, American Society of Plant Biologists; Department of Plant Sciences and Genome Center, University of California, Davis, CA 95616, USA

Plants employ a stunning variety of mechanisms to attract pollinators. For example, did you know that some plants generate heat and regulate their body temperature like mammals do to entice pollinators? The sacred lotus (*Nelumbo nucifera*) exhibits this remarkable trait. Incredibly, lotus flowers are capable of maintaining a stable internal temperature of 30 to 35 °C for 2 to 4 days while blooming, despite surrounding temperatures fluctuating between 10 and 30 °C (Seymour and Schultze-Motel 1996). Floral thermogenesis is driven by elevated respiration through the alternative oxidase (AOX) pathway (Watling et al. 2006), consuming lots of stored starch and lipids in the lotus flower. Thermogenesis benefits both the lotus plant and its pollinators, enhancing the emission of volatile organic compounds to attract insects, supporting pollen tube growth for successful fertilization, and offering pollinator insects, many of which are not capable of generating heat, warmth, and nutrition themselves.

The lotus flower contains both female and male reproductive organs, with the female organs (pistils), assembled as a central receptacle, maturing a day before the male organs (stamens). This sequential development facilitates outcrossing through insect pollinators. Under natural growth conditions, thermogenesis initiates when the tips of the unopened flower petals turn pink. Early morning the following day, as the petals begin to open, the flower emits heat and scent, signaling that the pistils are ready to receive pollen. By noon, the petals close again, forming an insulated chamber that retains warmth. Pollinators carrying pollen from other flowers may reside overnight. The next morning, as the flower's own pollen has matured, the petals reopen to release the insects to pollinate other receptive flowers (Seymour and Schultze-Motel 1996; Nagai et al. 2022). Given its metabolic cost (Grant et al. 2008, 2010), floral thermogenesis must be regulated. This raises fundamental questions: How is heat generation spatially and temporally controlled? Which tissues are responsible, and what molecular signals are involved?

In this issue of *Plant Physiology*, Yu et al. (2025b) applied a multiscale imaging approach to map thermogenic tissues in the *Nelumbo nucifera* flowers. First, the authors generated a morphological framework describing lotus receptacle development. This was used to inform imaging experiments. Traditionally, the gold standard for measuring temperature is a thermocouple (Lamprecht et al. 1998; Grant et al. 2008). A thermocouple works like a metal probe in a kitchen food thermometer, as it measures temperature by detecting how heat changes electrical voltage at the tip, whereas infrared (IR) thermal imaging provides spatial visualization of heat distribution mainly on a surface. Previous studies using IR thermal imaging suggested that both stamens and receptacles are thermogenic tissues (Grant et al. 2010). Conversely, IR imaging by Yu et al. (2025b) revealed that the epidermis and carpels of the receptacle were 4 to 5 °C warmer than the receptacle's inner porous structures or the innermost layer of petals.

To probe the biochemical basis of this localized heating, the authors examined the spatial distributions of elements using micro-X-ray fluorescence imaging. Potassium appeared evenly distributed, while calcium ions (Ca²⁺) were enriched in thermogenic zones. Ca²⁺-specific staining confirmed that epidermal cells in thermogenic receptacles exhibited a 4-fold increase in Ca²⁺ near the plasma membrane compared to pre-thermogenic stages. Furthermore, non-invasive micro-test techniques showed that Ca²⁺ influx rates were also 4 times higher at the onset of thermogenesis, implicating calcium as a key regulatory signal.

A published transcriptomic dataset describing gene expression at multiple stages in lotus thermogenesis was interrogated to investigate the expression of genes associated with Ca^2+^ transport (Yu et al. 2025a). *Mitochondrial Calcium Uniporter* (*MCU*) genes are universal regulators of intracellular Ca^2+^ signaling (Teardo et al. 2016). The highest expressed *MCU* gene had significantly elevated expression at the initiation and peak of thermogenesis. Mitochondria serve as both regulators and targets of Ca²⁺, using it to modulate metabolic activity. Yu et al. (2025b) also observed upregulation of Ca^2+^ ATPases and cation exchangers, suggesting an active buffering system to prevent cytosolic Ca^2+^ toxicity. Notably, genes involved in the tricarboxylic acid cycle were also elevated, linking Ca^2+^ influx to enhanced respiration (Figure).

**Figure.**
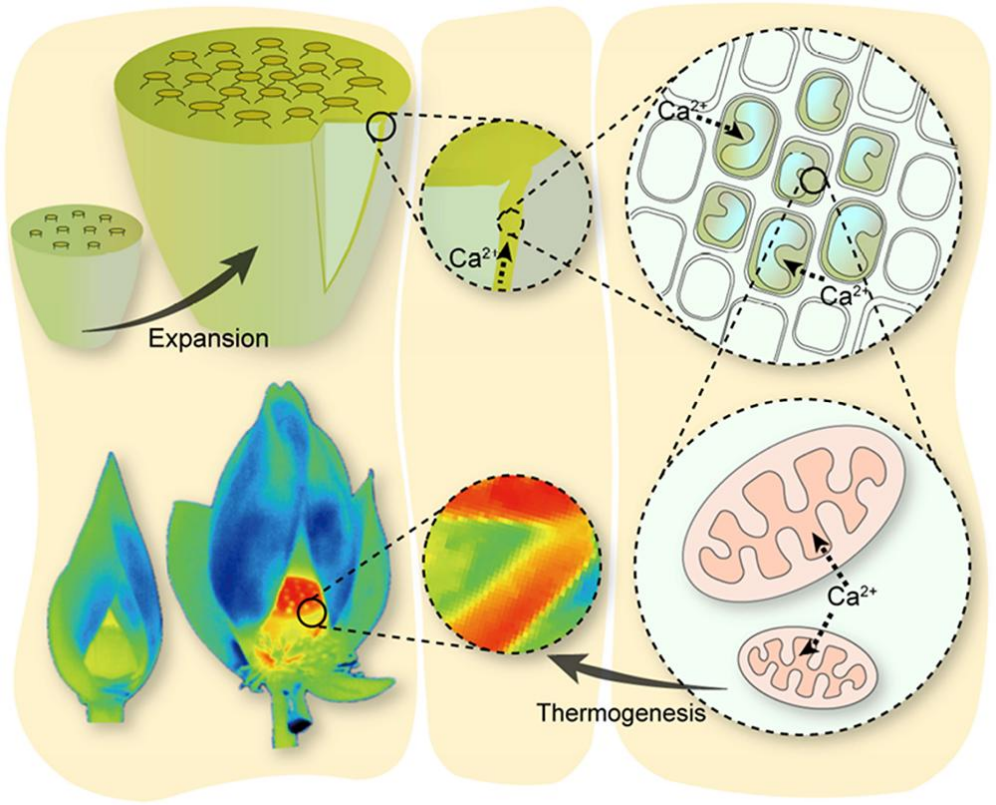
Proposed model of thermogenesis in *Nelumbo nucifera*. Figure from Yu et al. (2025a). The lotus receptacle expands at the initiation of thermogenesis. Ca²⁺ ions are transported to the receptacle and enter epidermal cells through calcium channels in the apoplast. An influx of Ca²⁺ occurs in the cytosol, triggering mitochondrial respiratory regulation and promoting thermogenesis.

In plant mitochondria, respiration is facilitated by 2 primary terminal oxidases: cytochrome *c* oxidase (COX) and AOX. COX functions as the main pathway, efficiently producing ATP, while AOX bypasses ATP generation and releases heat (Hoefnagel et al. 1995; Ribas-Carbo et al. 1995). In lotus, AOX showed substantial activity at the onset of thermogenesis by branching electrons from COX (Watling et al. 2006; Grant et al. 2008). Application of the AOX inhibitor salicylhydroxamic acid significantly reduced oxygen consumption, confirming high alternative respiration rates in receptacles. When salicylhydroxamic acid was combined with ionomycin, a Ca^2+^ ionophore that increases cytosolic Ca^2+^, total respiration briefly increased, suggesting that Ca^2+^ promotes alternative respiration. Interestingly, ionomycin alone inhibited respiration, though EDTA, a Ca^2+^ chelator, paradoxically increased total respiration. These results suggest a complex role for cytosolic Ca^2+^ in balancing cytoplasmic and alternative respiration.

In conclusion, this study employs complementary imaging techniques that illuminate thermogenesis in *N. nucifera* at both an organ and tissue scale. IR thermal imaging reveals heating occurs specifically in the carpels and epidermis, and micro-X-ray fluorescence imaging suggests Ca^2+^ ions are increased in these tissues. Although further development of non-invasive or high-resolution imaging, at the cellular level, is needed, and the precise molecular mechanisms remains to be fully clarified, the authors link thermogenesis to increased Ca^2+^ and respiration levels. In the future, emerging technologies such as spatial transcriptomics may provide deeper insights at a microscopic scale. Ultimately, a more comprehensive understanding of how lotus flowers precisely regulate heat production will not only advance our knowledge of plant reproductive physiology but will also shed light on broader themes in plant–environment interactions and evolutionary adaptation.

## Data Availability

No new data were generated or analyzed in support of this research.
