# Correction to: Thermogenic tissues in lotus: Insights from multiscale imaging and calcium dynamics

**DOI:** 10.1093/plphys/kiaf307

**Published:** 2025-07-29

This is a correction to: Erin Cullen, Chong Teng, Thermogenic tissues in lotus: Insights from multiscale imaging and calcium dynamics, *Plant Physiology*, Volume 198, Issue 1, May 2025, https://doi.org/10.1093/plphys/kiaf173

In the originally published version of the manuscript, in the sixth paragraph, the second sentence: “COX functions as the main pathway, efficiently producing ATP whilst AOX bypasses ATP generation but releases heat and oxygen[…]” is emended to read instead: “COX functions as the main pathway, efficiently producing ATP while AOX bypasses ATP generation and releases heat[…]”.

